# Robust and persistent SARS-CoV-2 infection in the human intestinal brush border expressing cells

**DOI:** 10.1080/22221751.2020.1827985

**Published:** 2020-10-03

**Authors:** Sunhee Lee, Gun Young Yoon, Jinjong Myoung, Seong-Jun Kim, Dae-Gyun Ahn

**Affiliations:** a Center for Convergent Research of Emerging Virus Infection, Korea Research Institute of Chemical Technology, Daejeon, South Korea; b Korea Zoonosis Research Institute & Genetic Engineering Research Institute, Jeonbuk National University, Jeollabuk-do, South Korea

**Keywords:** COVID-19, SARS-CoV-2, coronavirus, persistent, ACE2

## Abstract

Studies on patients with the coronavirus disease-2019 (COVID-19) have implicated that the gastrointestinal (GI) tract is a major site of severe acute respiratory syndrome coronavirus 2 (SARS-CoV-2) infection. We established a human GI tract cell line model highly permissive to SARS-CoV-2. These cells, C2BBe1 intestinal cells with a brush border having high levels of transmembrane serine protease 2 (TMPRSS2), showed robust viral propagation, and could be persistently infected with SARS-CoV-2, supporting the clinical observations of persistent GI infection in COVID-19 patients. Ectopic expression of viral receptors revealed that the levels of angiotensin-converting enzyme 2 (ACE2) expression confer permissiveness to SARS-CoV-2 infection, and TMPRSS2 greatly facilitates ACE2-mediated SARS-CoV-2 dissemination. Interestingly, ACE2 but not TMPRSS2 expression was significantly promoted by enterocytic differentiation, suggesting that the state of enterocytic differentiation may serve as a determining factor for viral propagation. Thus, our study sheds light on the pathogenesis of SARS-CoV-2 in the GI tract.

## Introduction

A novel coronavirus, severe acute respiratory syndrome coronavirus 2 (SARS-CoV-2) emerged in Wuhan, China, in December 2019, and since then it has rapidly spread worldwide threatening global public health [1,2]. Coronavirus Disease-19 (COVID-19) caused by SARS-CoV-2, leads to fever, cough, myalgia, fatigue, diarrhea, pneumonia, and even death in severe cases [1]. To date (July, 2020), there have been over 16 million confirmed cases of SARS-CoV-2 infection, and more than 650,000 deaths worldwide [3].

Coronaviruses are a large family of viruses that cause respiratory diseases, gastroenteritis, hepatitis, and encephalitis in animals and humans [4]. They consist of a large, single-stranded, positive-sense RNA with a 5′ untranslated region (UTR), 6–10 open reading frames (ORFs), and a 3′ UTR. ORF 1a and 1b encode two replicase polyproteins, polyprotein 1a (pp1a) and polyprotein 1ab (pp1ab), which are further processed by viral proteases to produce various nonstructural proteins. The other ORFs encode four major structural proteins, spike (S), envelope (E), membrane (M), and nucleocapsid (N), and several other accessory proteins [4]. Among these viral structural proteins, the S glycoprotein is responsible for cellular receptor binding and membrane fusion during viral entry into the host cell. Moreover, it consists of two subunits, S1 and S2; the S1 subunit contains a receptor-binding domain (RBD) that recognizes and interacts with cellular receptors, and the S2 subunit mediates membrane fusion [5–8].

The entry of SARS-CoV-2 into the host cell depends mainly on two receptors, angiotensin-converting enzyme 2 (ACE2) and type II transmembrane serine protease (TMPRSS2) [9–12]. Several other receptors and proteases such as DC-SIGN, L-SIGN, Neuropilin-1, furin, TMPRSS4, and cathepsin B and L, are also associated with the viral entry [11–16]. ACE2 serves as one of the major determinants of tissue tropism by binding to SARS-CoV-2 via the RBD of the S protein [11]. On the other hand, TMPRSS2 activates the S protein by proteolytic priming and facilitates entry into the host cell. Furthermore, the cleavage of S protein by host proteases at the S1/S2 multibasic and S2′ sites is essential for viral infectivity [11,17,18]. In Middle East respiratory syndrome coronavirus (MERS-CoV) infection, the cleavage of the S2′ site by TMPRSS2 and the S1/S2 sites by furin protease is essential for efficient viral entry into the host cell, depending on the cell type [19]. Further, ACE2 undergoes a proteolytic process by TMPRSS2 that promotes viral entry into the host cells during SARS-CoV infection [20]. DC-SIGN (CD209) and L-SIGN (CD209L), immunoglobulin-like cell adhesion molecule superfamily receptors, and Neuropilin-1 can be alternative receptors that SARS-CoV-2 binds to in the vascular and nervous systems, respectively. L-SIGN serves as a receptor and attachment factor for several viruses, including SARS-CoV, HCoV-229E, HIV, Ebola, and other enveloped viruses [13,21–23]. DC-SIGN and L-SIGN can bind to the RBD of S proteins, facilitating SARS-CoV-2 entry. However, the binding affinity of ACE2 to RBD is higher than that of DC-SIGN and L-SIGN [13]. SARS-CoV-2 can also utilize endosomal protease cathepsin B and L during entry via endocytosis [11,15].

The distribution of these receptors in human tissues is highly correlated with the infection sites of SARS-CoV-2, particularly in the epithelia of lung and gastrointestinal tract [24,25]. Thus, it is important to study the precise relationship between viral propagation and receptor expression levels in those infection sites. In this study, we investigated the permissiveness of various human epithelial cell lines to SARS-CoV-2 infection and the virus growth kinetics in conjunction with receptor expression levels. We further characterized receptor expression during viral infection or cellular differentiation. Our findings, including the association of ACE2 and dipeptidyl peptidase 4 (DPP4) expression with enterocytic differentiation, will be useful for understanding coronavirus pathogenesis in the gastrointestinal tract.

## Materials and methods

### Cell culture and virus infection

Vero (ATCC CCL-81) and A549 (ATCC CCL-185) cells were maintained in Dulbecco’s Modified Eagle medium with high glucose (DMEM, HyClone, Logan, UT, USA) with 10% fetal bovine serum (FBS; Invitrogen, Grand Island, NY, USA) and 1% penicillin/streptomycin (Invitrogen, Grand Island, NY, USA). Calu-3 (ATCC HTB-55), Caco-2 (ATCC HTB-37), C2BBe1 (ATCC CRL-2102), and RPMI 2650 (ATCC CCL-30) cells were maintained in Modified Eagle medium (MEM) with Earle’s Balanced Salt Solution (MEM/EBSS, HyClone) supplemented with 10% FBS and 1% penicillin/streptomycin. PK-15 (ATCC CCL-33), IPEC-J2, and NCI-H292 (ATCC CRL-1848) cells were grown in RPMI 1640 medium (Invitrogen, Grand Island, NY, USA) containing 10% FBS and 1% penicillin/streptomycin. The cells were maintained at 37 °C in a humidified atmosphere containing 5% CO_2_. Cells were seeded on 48-well or 6-well tissue culture plates and infected with SARS-CoV-2. After 1 h incubation, cells were washed with media to remove the remaining virus and incubated with fresh media.

The pathogen resources (NCCP43326) and SARS-CoV-2 (BetaCoV/Korea/KCDC03/2020) for this study were provided by the National Culture Collection for Pathogens [26]. All the experiments with the infectious virus were conducted in a biosafety level 3 (BSL-3) laboratory at the Korea Research Institute of Chemical Technology (KRICT).

### Ectopic expression of ACE2 and TMPRSS2

Human ACE2 and TMPRSS2 plasmids were purchased from Sino Biological, Beijing, China. Cells grown on a 48-well plate were transiently transfected with hACE2 or hTMPRSS2 expressing plasmids. After 24 h, the cells were infected with 1 MOI of SARS-CoV-2 and incubated for 1 h. The cells were then washed with media and incubated for 48 h. Viral RNAs were extracted from the media collected at 48 dpi.

### Differentiation of C2BBe1 cells

Differentiation of C2BBe1 was performed as previously described by Huang et al. [27]. Briefly, the C2BBe1 cells were seeded on trans-well inserts (0.4 µm pore size, 12-mm membrane diameter; Corning, Kennebunk, ME, USA) at a density of 5 × 10^5^ cells/cm^2^ and cultured in 1:1 ratio of MEM/EBSS containing 10% FBS: enterocyte differentiation medium (Corning, Kennebunk, ME, USA). After 24 h, the medium was changed to enterocyte differentiation medium supplemented with 1% ITS-A (Invitrogen, Grand Island, NY, USA) and maintained for at least 5 days.

### Persistent infection in C2BBe1 cells

C2BBe1 cells seeded on a 6-well tissue culture plate were infected with 1 MOI of SARS-CoV-2 and incubated for 1 h. After washing with MEM/EBSS, the cells were incubated with MEM/EBSS containing 10% FBS and 1% penicillin/streptomycin. After initial infection for 3 days, the cells were serially passaged 4 times at 5-day intervals using 0.25% trypsin-EDTA (Invitrogen, Grand Island, NY, USA). The culture supernatants were collected at each passage for quantification of viral RNA levels.

### Quantitative real-time RT–PCR

Viral RNA was extracted from cell culture supernatants using the QIAamp Viral RNA mini kit (Qiagen, Hilden, Germany) according to the manufacturer’s protocol. For the detection of SARS-CoV-2, the N gene target of the 2019-nCoV CDC RUO kit (IDT, Coralville, IA, USA) was used. Quantitative real-time RT–PCR was performed with the One Step PrimeScript III RT-qPCR mix (Takara, Shiga, Japan) using the QuantStudio 3 Real-Time PCR system (Life Technologies, Carlsbad, CA, USA). Total cellular RNA was isolated using the RNeasy Mini kit (Qiagen, Hilden, Germany) according to the manufacturer’s protocols. cDNA was synthesized using the Superscript IV first-strand synthesis kit for RT–PCR (Invitrogen, Grand Island, NY, USA) according to the manufacturer’s protocol. Quantitative real-time PCR was performed using LaboPass SYBR Green Q master mix (Cosmogenetech, Seoul, South Korea) on a QuantStudio 3 Real-Time PCR system (Life Technologies). For real-time PCR, the following primers were used: human ACE2 forward 5´-TACAGTACTGGAAAAGTTTG-3´ and reverse 5´-CCTCACATAGGCATGAAGATG-3´, human TMPRSS2 forward 5´-TTCATCCTTCAGGTGTACTCATCT-3´ and reverse 5´-CTATACAGCGTAAAGAAACCACT-3´, human IFN-α1 forward 5´-ATGGCCTCGCCCTTTGCTTTA-3´ and reverse 5´-TTTCTGCTCTGACAACCTCCC, human IFN-α2 forward GGCTGAAACCATCCCTGTCC-3´ and reverse 5´-CTCCCAGGCACAAGGGCTG-3´, human IFN-β 5´-forward GATTCATCTAGCACTGGCTGG-3´ and reverse 5´-CTTCAGGTAATGCAGAATCC-3´, human IFN-γ forward 5´-TGACCAGAGCATCCAAAAGAGTG-3´ and reverse 5´-CAGCATCTGACTCCTTTTTCGC-3´, human IFN-λ1 forward 5´-TTGCAGCTCTCCTGTCTTCC-3´ and reverse 5´-CCAGGACCTTCAGCGTCAG-3´, human IFN-λ3 forward 5´- CATAGCCCAGTTCAAGTCCC-3´ and reverse 5´-CACTTGCAGTCCTTCAGCAG-3´, intestinal alkaline phosphatase (IAP) forward 5´-AGTTATCCTGCTCCCCACCT-3´ and reverse 5´-TAGGA-GGTGAAGGTCCAACG-3´, sucrose-isomaltase (SI) forward 5´-GTGGCTGCTAACATCCCCTA-3´ and reverse 5´-GAGGAAGGTCCTGGAATGCT-3´, human DPP4 forward 5´-ATTCAATATCTCCTGATGGGCAGT-3´ and reverse 5´-CACTAAGCAGTTCCATCTTCCAC-3´, and human GAPDH forward 5´-GGAGCGAGATCCCTCCAAAAT-3´ and reverse 5´-GGCTGTTGTCATACTTCTCACGG-3´. The mRNA expression levels were normalized to human GAPDH mRNA, and the relative quantities were calculated by the 2^-ΔΔCt^ method.

### Western blot analysis

The cells were harvested and lysed using lysis buffer (20 mM Tris-HCl [pH 7.5], 150 mM NaCl, 1 mM EDTA, 50 mM NaF, 1 mM Na_3_VO_4_, 0.5% Triton X-100, 10% SDS, and 1 mM β-glycerophosphate) supplemented with 1% protease inhibitor cocktail (Thermo Fisher Scientific). The following antibodies were used for the detection of the cellular or viral proteins: anti-ACE2 antibody (Abcam, Cambridge, UK), anti-SARS-CoV-2 nucleocapsid (N) protein antibody (Sino Biological, Beijing, China), anti-DPP4 (Abcam), anti-GAPDH (Cell Signaling Technology, Danvers, MA, USA), and anti-β-actin (Cell Signaling Technology). The blots were incubated with the appropriate HRP-conjugated secondary antibodies (Cell Signaling Technology). The membranes were developed with ECL solution (PerkinElmer, Waltham, MA, USA) and imaged using ImageQuant Las 4000 (GE Healthcare, Freiburg, Germany). The intensities of the protein bands were quantified using ImageJ software (National Institutes of Health, Bethesda, Maryland, USA) [28]. Data are presented as mean values with error bars showing the standard deviations from three independent experiments unless otherwise specified.

### Statistical analysis

Pearson’s correlation coefficients (Pearson’s r) were calculated with a two-tailed Pearson test using GraphPad Prism 8. *P* values of less than 0.05 were considered statistically significant.

## Results

### TMPRSS2 exploits ACE2-mediated SARS-CoV-2 dissemination

**Figure 1.**
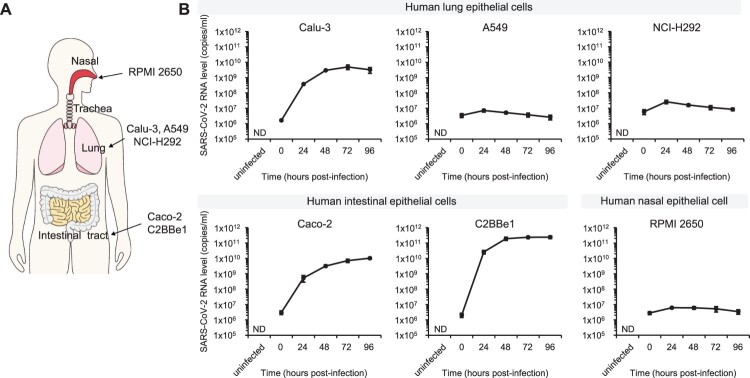
The levels of SARS-CoV-2 titer in human epithelial cell lines corresponding to viral infection sites. (A) Schematic diagram of human epithelial cell lines corresponding to major infection sites of SARS-CoV-2. (B) The indicated cells were grown on a 48-well plate and infected with 5 MOI of SARS-CoV-2. Viral RNA levels were determined in the media collected at the indicated time points. Data are presented as mean values with error bars showing the standard deviations from three independent experiments. ND, not detected.

Passaging SARS-CoV-2 in the Vero cells (African green monkey kidney cells) multiple times may lead to mutations in the S protein associated with selective pressure in humans, hence, it is crucial to establish a novel human cell culture system for SARS-CoV-2 [29]. First, we examined the growth of SARS-CoV-2 in various human epithelial cell lines derived from major infection sites (nasal cavity, lungs, and intestine), namely, A549 (alveolar epithelial cells), Calu-3 (lung/bronchial epithelial cells), NCI-H292 (airway epithelial cells), RPMI 2650 (nasal epithelial cells), Caco-2 (colorectal epithelial cells), and C2BBe1 (sub-clone of Caco-2) (Figure 1A). Non-human epithelial cells were also included; Vero CCL81 cells (African green monkey kidney cells), PK-15 (porcine kidney cells), and IPEC-J2 (porcine intestinal cells) (Fig. S1). Vero CCL81 cells were used as the positive control. Among human cell lines, the Calu-3, Caco-2, and C2BBe1 cell lines were highly permissive to SARS-CoV-2 with the highest titer in the C2BBe1 cells (Figure 1B). Moreover, the PK-15 cells were also highly permissive to SARS-CoV-2 (Fig. S1). In contrast, SARS-CoV-2 did not propagate in the A549, NCI-H292, and RPMI 2650 cells. Significant cytopathic effects (CPEs) have been observed only in Vero cells. Mild CPEs were observed in Calu-3 and PK-15 as opposed to other human cell lines that did not show any CPEs (Fig. S2). The data obtained from the various cell lines are summarized in Table 1.

**Table 1.** Various cell lines tested for permissiveness to SARS-CoV-2 infection.

Cell line	Cell type	Susceptibility	CPE	Relative ACE2 level	Relative TMPRSS2 level
Vero CCL81	Monkey kidney epithelial cell	++++	++++	N/T*	N/T
A549	Adenocarcinomic human alveolar basal epithelial cell	−	−	−	−
Calu-3	Nonciliated human lung/bronchial epithelial cell	+++	++	++++	+++
Caco-2	Human epithelial colorectal adenocarcinoma cell	+++	−	+	+++
C2BBe1	Caco-2 sub-clone	++++	−	++	++++
RPMI 2650	Human nasal epithelial cell	−	−	+	+
NCI-H292	Human airway epithelial cell	+	−	++	+
PK-15	Porcine kidney epithelial cell	+++	++	N/T	N/T
IPEC-J2	Porcine intestinal columnar epithelial cell	++	−	N/T	N/T

*N/T: Not tested.

**Figure 2.**
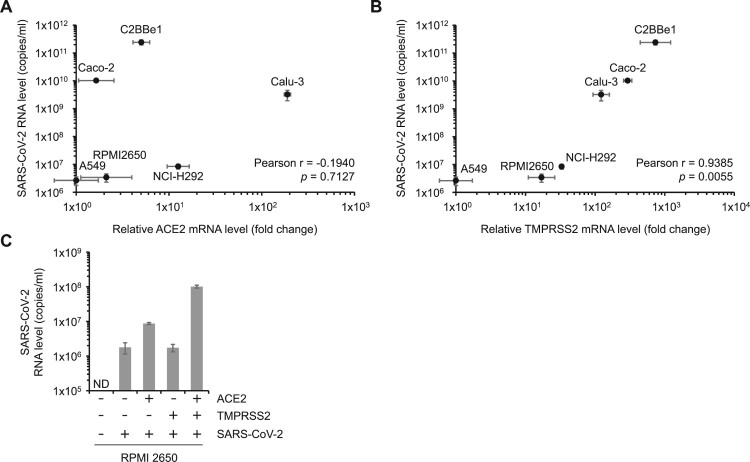
TMPRSS2 exploits ACE2-mediated SARS-CoV-2 dissemination. (A, B) The levels of SARS-CoV-2 titer and viral receptor expression were compared in various human epithelial cell lines. Viral RNA levels were plotted against relative mRNA levels of ACE2 (A) or TMPRSS2 (B). Relative ACE2 or TMPRSS2 mRNA levels represent the fold changes in the indicated mRNA level of each cell compared to A549 cells. Pearson coefficients (r) and *p*-values (*p*) were calculated as described in the materials and methods section. (C) ACE2 and TMPRSS2 were ectopically expressed in RPMI 2650 cells by transient transfection of ACE2 or TMPRSS2 expressing plasmids. After 24 h, the cells were infected with 1 MOI of SARS-CoV-2. Viral RNA levels in the media collected at 48 dpi were determined. ND, not detected.

Given the role of ACE2 and TMPRSS2 as SARS-CoV-2 receptors, we investigated the relationship between the receptor expression levels and viral propagation in human epithelial cells. The viral titer was compared with the basal mRNA levels of ACE2 and TMPRSS2 in each cell line. Since the A549 cells had the lowest levels of both the receptors, the relative gene expression levels of ACE2 and TMPRSS2 in the other cell lines were compared to those of A549 cells. The levels of the ACE2 protein in each cell line were well correlated with its mRNA levels (Figure 2A and Fig. S3). ACE2 protein was not detected by Western blot analysis in A549 cells (Fig. S3). The viral titers were plotted against the mRNA levels of ACE2 or TMPRSS2 (Figure 2A and 2B). While the correlation of the ACE2 mRNA levels with the viral RNA levels were not significant (Pearson’s correlation coefficient *r *= −0.1940, *p *= 0.7127) (Figure 2A), the TMPRSS2 mRNA levels showed strong correlation (Pearson’s correlation coefficient *r *= 0.9385, *p *= 0.0055) in the human epithelial cell lines (Figure 2B). These data suggest that although ACE2 is necessary for SARS-CoV-2 infection, it may not be the only factor affecting the viral propagation. This also suggests the importance of the coordination between ACE2 and TMPRSS2 expression levels in the process of viral propagation.

To determine the effects of ACE2 and TMPRSS2 levels on SARS-CoV-2 pathogenesis, we investigated whether the ectopic expression of ACE2 and TMPRSS2 can enhance viral dissemination. We ectopically expressed the two receptors in RPMI 2650 cells expressing levels of ACE2 similar to the Caco-2 cells but not permissive to SARS-CoV-2. The ectopic expression of ACE2 allowed the cells to be permissive to SARS-CoV-2, showing viral RNA levels 4.9 times higher than that in the empty vectors (Figure 2C). In contrast, TMPRSS2 alone did not enhance viral infectivity even in the presence of ACE2. It is thus highly likely that TMPRSS2 alone does not increase permissiveness to SARS-CoV-2 when the ACE2 levels are not sufficient for viral infection. However, the co-expression of ACE2 and TMPRSS2 significantly enhanced viral dissemination by 56.7 times compared to that of the empty vector. These results suggest that ACE2 levels confer permissiveness to SARS-CoV-2 depending on the cell types and that TMPRSS2 greatly accelerates ACE2-mediated viral propagation. This may explain the robust viral propagation in the Caco-2 and C2BBe1 cells having high levels of TMPRSS2, despite the low levels of ACE2.

### Expression of ACE2, but not TMPRSS2, is dynamic and is associated with the intestinal epithelial cell differentiation

**Figure 3.**
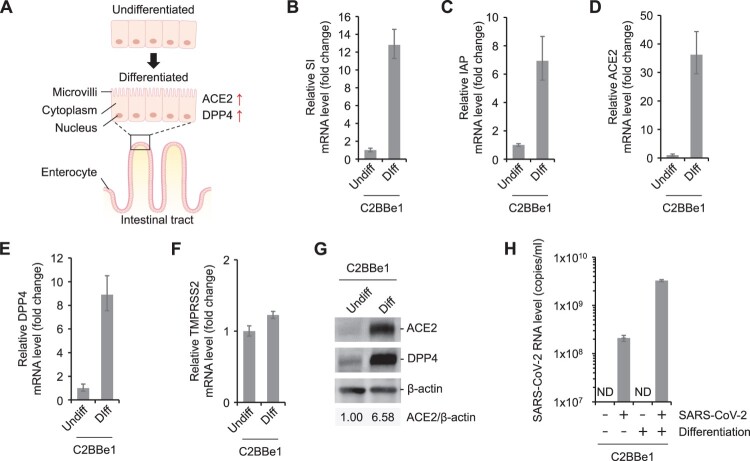
Dramatic induction in ACE2 and DPP4 expression by cellular differentiation. (A) Schematic diagram of the structure of enterocytes. (B-F) C2BBe1 cells were differentiated on trans-well filters. The undifferentiated cells were seeded on a day before harvesting. The mRNA levels of the indicated genes normalized to GAPDH in the undifferentiated (Undiff) and differentiated cells (Diff) were compared. SI, sucrose-isomaltase; IAP, intestinal alkaline phosphatase. (G) The protein expression levels of ACE2 and DPP4 were detected in the cell lysates from the undifferentiated and differentiated C2BBe1 cells by Western blotting. The ratio of ACE2 to β-actin was determined by densitometric analysis. (H) The undifferentiated and differentiated C2BBe1 cells were infected with 0.2 MOI of SARS-CoV-2. The viral titer in the media was determined at 2 dpi. ND, not detected.

Here, we noted that the levels of SARS-CoV-2 titers were higher in the C2BBe1 cells compared to the parental Caco-2 cells (Figure 1B). C2BBe1 cells, having brush borders with microvilli resembling the brush border of human intestinal epithelia, were selected from the parental Caco-2 cell line [30]. The genetic backgrounds of the C2BBe1 cells are very close to that of the human intestinal epithelial cells [31]. Moreover, these cells have a more homogeneous brush border expression and contain more microvillar proteins than the parental Caco-2 cells [30]. C2BBe1 cells can be further differentiated by the growth of the trans-well filters inducing structure of the microvilli (Figure 3A) [27,30]. Thus, we examined whether cellular differentiation can regulate the expression of these two receptors (ACE2 and TMPRSS2). C2BBe1 cells were differentiated by growth on the trans-well filters for at least 5 days. The undifferentiated C2BBe1 cells were seeded on the trans-well filters a day before the experiments. Gene expression of intestinal alkaline phosphatase (IAP) and sucrose-isomaltase (SI) were used as cellular differentiation markers. Previously, biochemical analysis revealed that the enterocytic differentiation of Caco-2 cells correlates well with the intestinal alkaline phosphatase (IAP) and sucrose-isomaltase (SI) gene expression [32–35]. IAP and SI mRNA levels in the differentiated C2BBe1 cells significantly increased compared to the undifferentiated cells, confirming the enterocytic differentiation of C2BBe1 cells on the trans-well filters (Figure 3B and 3C). Interestingly, the ACE2 mRNA levels greatly increased in the differentiated C2BBe1 cells (Figure 3D). The induction of ACE2 protein was also confirmed by Western blot analysis (Figure 3G). This suggests the possibility of SARS-CoV-2 infection to the gastrointestinal (GI) tract through enterocytes, where ACE2 expression is abundant. Since MERS-CoV can infect the GI tract [36], we also examined the levels of the human dipeptidyl peptidase 4 (DPP4), a receptor for MERS-CoV, in the differentiated C2BBe1 cells. Similar to the ACE2 receptor, both mRNA and protein levels of DPP4 increased drastically by cellular differentiation (Figure 3E and 3G). This is consistent with previous observations that the DPP4 protein levels correlate with the stage of enterocytic differentiation [35]. In contrast to ACE2 and DPP4, the TMPRSS2 mRNA levels were comparable in the undifferentiated and differentiated cells (Figure 3F). Next, we evaluated whether the cellular differentiation-inducing ACE2 receptor can promote viral propagation. Cells were infected with low MOI (0.2 MOI) of SARS-CoV-2 to avoid saturation of SARS-CoV-2 titer. SARS-CoV-2 titer was 15.6 times higher in the differentiated cells than in the undifferentiated cells (Figure 3H). Hence, our data suggest that the state of enterocytic differentiation may serve as a determinant factor of viral propagation inducing dramatic ACE2 and DPP4 expression, but not TMPRSS2 (Figure 3A). In other words, enterocytes expressing brush borders and microvilli, prevalent with viral receptors, will provide a favourable environment for recently emerging coronaviruses such as SARS-CoV, SARS-CoV-2, and MERS-CoV.

**Figure 4.**
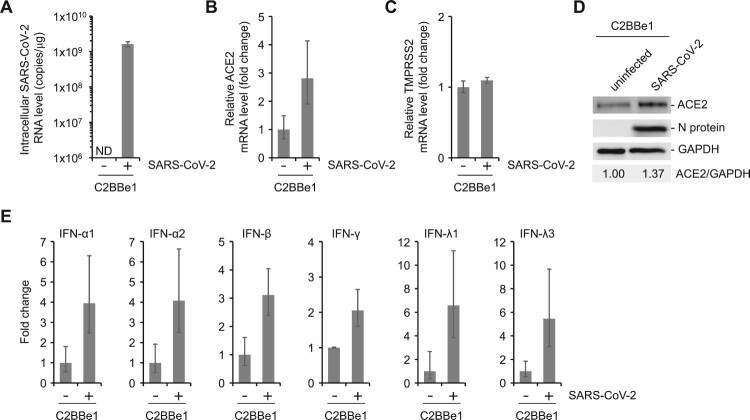
Increased expression of ACE2 receptor in C2BBe1 cells during SARS-CoV-2 infection. (A-C) C2BBe1 cells were infected with 1 MOI of SARS-CoV-2. The mRNA levels of the indicated genes were measured from the cells harvested at 72 hpi. Each mRNA level was normalized to that of GAPDH. (D) ACE2 and SARS-CoV-2 nucleocapsid (N) protein in the C2BBe1 cells with or without viral infection were detected by Western blotting. The ratio of ACE2 to GAPDH was determined by densitometric analysis. (E) The mRNA levels of the indicated interferon genes were measured. Each mRNA level was normalized to that of GAPDH.

Since the ACE2 receptor is known to be an interferon-stimulated gene [37], we analyzed whether these receptors can be affected by the viral infection. The ACE2 levels were measured in the presence or absence of SARS-CoV-2 infection. A significant amount (1.6 × 10^9^ copies/μg) of intracellular viral RNA was detected only in the infected cells (Figure 4A). The N protein of SARS-CoV-2 was detected in the infected cells but not in the uninfected cells (Figure 4D). The expression levels of the ACE2 gene were higher in the infected cells compared to the uninfected cells (Figure 4B). The increase in the ACE2 protein levels was further confirmed by Western blotting and densitometric analysis (Figure 4D). The ratio of ACE2 protein level to GAPDH was 1.37 times higher in the SARS-CoV-2 infected cells than that in the uninfected cells (Figure 4D). In contrast, the TMPRSS2 mRNA levels remained unchanged (Figure 4C). IFN-α1, α2, β, γ, λ1, and λ3 levels were also analyzed following viral infection (Figure 4E). Overall, the IFN responses were enhanced in the SARS-CoV-2 infected cells. However, given that the basal level of ACE2 mRNA level are highly dynamic depending on the cell lines or cellular differentiation (Figure 2A and 3D), the increase in ACE2 level by IFN responses during infection seems rather limited.

### Persistent infection with SARS-CoV-2 in C2BBe1 cells

**Figure 5.**
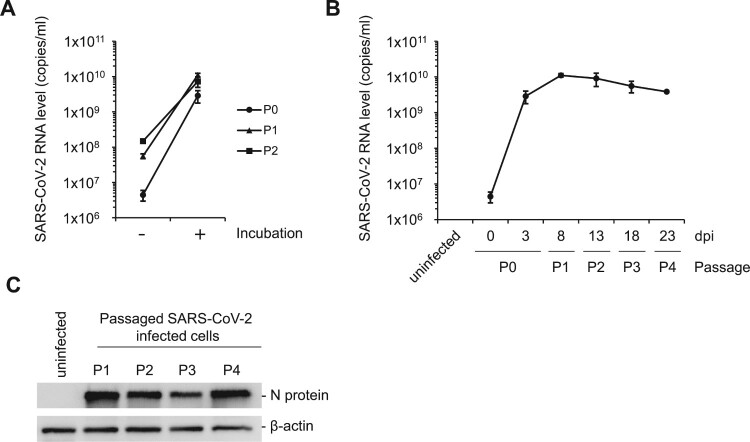
Persistent infection of SARS-CoV-2 in C2BBe1 cells. Initially, 1 MOI of SARS-CoV-2 was infected into C2BBe1 cells (P0). The cells were passaged 4 times (P1-P4) for a total of 23 days. (A) At each indicated passage, fresh media was added and the cells were grown until 70–80% confluency. Media were collected before and after incubation. Viral titers in the collected media were quantified. (B) Viral titers in the media collected at each passaging was determined. (C) SARS-CoV-2 nucleocapsid (N) protein was detected by Western blotting using the total cell lysates from the indicated passage. Data are presented as mean values with error bars showing the standard deviations from three independent experiments.

Since the viral growth increased continuously in the C2BBe1 cells without any cytopathic effects (Figure 1B and Fig. S2), we asked whether SARS-CoV-2 can persistently infect these cells. Initially, the C2BBe1 cells were infected with 1 MOI of SARS-CoV-2 (P0). After 3 dpi, the infected C2BBe1 cells were washed with media and passaged serially with an additional 4 times (P1-P4) (Fig. S4A). At initial infection (P0), viral RNA levels of the media collected after 3 dpi significantly increased as compared to that of media collected at the time of infection (Figure 5A). Similarly, after first (P1) and second (P2) passaging, increased viral RNA levels were observed in the media collected before passaging, suggesting the accumulation of progeny viruses generated from the infected cells (Figure 5A). Further, we measured the levels of the accumulated progeny viruses in the media after each passaging. The progeny virus production of SARS-CoV-2 from the infected cells was maintained at a high level (>10^9^ genome copies/ml) for at least 23 days (Figure 5B). The levels of the SARS-CoV-2 N protein in the infected cells were also compared after each passaging. The levels of N protein were similar in all the passaged cells (P1-P4) (Figure 5C). In contrast, the N protein was not detected in the uninfected cells. Moreover, we examined the infectivity of the progeny viruses after prolonged passaging. 100 microliters of the media after the fourth passaging (P4) were used for the reinfection of Vero CCL81 cells. Significant CPE was observed at 2 dpi, confirming the infectivity of the progeny viruses (Fig. S4B). Therefore, our data strongly suggest that SARS-CoV-2 is capable of establishing persistent infection in C2BBe1 intestinal epithelial cells.

## Discussion

In addition to a brush border protein pattern functionally similar to that of human intestinal enterocytes [30], the C2BBe1 cells dynamically express ACE2 and DPP4 receptors depending on the state of enterocytic differentiation. Hence, the C2BBe1 cells may serve as an important *in vitro* model cell line to study the pathogenesis of the SARS-CoV-2 in the gastrointestinal (GI) tract. Although the primary infection sites of SARS-CoV-2 are the airway and lung epithelia, several studies have revealed that the GI tract is another major infection site of SARS-CoV-2 [25,38]. Potential SARS-CoV-2 infection in the GI tract has also been shown in the human gut organoids developed from primary intestinal epithelial stem cells [39].

During the MERS-CoV and SARS-CoV outbreaks, 20–25% of the infected cases showed symptoms of GI infection [2]. Similarly, 25% of the COVID-19 patients show symptoms of GI infection such as diarrhea, and the fecal samples of 48–53% of the patients tested positive for viral RNA [40]. Moreover, prolonged shedding of SARS-CoV-2 has been observed in the stools or rectal swab samples. The stool samples of 23% of the patients continued to be tested positive for the virus even after obtaining negative results in the respiratory samples [25]. A recent study showed that the rectal swabs of 8 pediatric COVID-19 patients persistently tested positive after nasopharyngeal testing was negative, suggesting that the viral shedding from the GI tract might last longer than that from the respiratory tract [41]. In other cases, 3 COVID-19 patients with GI symptoms were re-admitted after pneumonia had resolved due to the persistence of intestinal SARS-CoV-2 infection [42]. Thus, our findings are in line with these clinical observations supporting the persistence of SARS-CoV-2 infection in the GI tract. The prevalence of ACE2 and DPP4 expression in the differentiated intestinal cells representing enterocytes also suggests that GI infection is a common feature of COVID-19.

Recent studies have revealed that ACE2 and TMPRSS2 receptors are essential for the SARS-CoV-2 entry into the host cells [11,14]. Despite the diverse tissue distribution of these receptors, many studies have shown their presence in the epithelia of the lung and GI tract. RNA-seq profile analysis showed that the ACE2 gene is highly expressed in the small intestine and enriched in epithelial cells with 93.38% ACE2-positivity [38,43]. Moreover, the ACE2 protein expression on the surface of the lung alveolar epithelial cells and enterocytes of the small intestine was confirmed by immunohistochemistry [24]. Particularly, the expression of ACE2 in the enterocytes is confined to the brush border [24]. The increase in the ACE2 levels by enterocytic differentiation in the C2BBe1 cells may explain this expression pattern of ACE2 in the enterocytes. Moreover, similar ACE2 induction by cellular differentiation has also been observed in human airway epithelia, suggesting that the strong expression of ACE2 in the brush border may not be restricted to the enterocytes [44].

The presence of TMPRSS2 protein in the human GI and respiratory tract tissues has been previously confirmed by immunohistochemistry [45,46]. The RNA-seq profile showed higher gene expression of TMPRSS2 in the primary human ileum enteroids compared to other cell lines (i.e. HEK293, Huh7.5, Hela, and HT-29) [14]. Moreover, the co-expression of ACE2 and TMPRSS2 in the GI tract has also been observed previously [37]. Thus, in the context of virus-receptor interactions, the brush border of enterocytes in the GI tract may provide a favourable environment for the SARS-CoV-2 infection. Persistent GI tract infection is also linked to possible fecal-to-oral transmission as an alternative route for viral spread. However, Zang et al. have previously shown that the infectivity of SARS-CoV-2 was reduced in the human GI tract due to the low pH of the gastric fluid, and they were unable to recover the virus from the stool samples of a small group of COVID-19 patients even though SARS-CoV-2 RNAs were detected in these samples [14]. Hence, the possibility of fecal-oral transmission should be carefully addressed in the future.

Because ACE2 expression can be induced by IFN or inflammation, there is an increased possibility of SARS-CoV-2 spread via immune responses [37]. Although we observed an enhanced ACE2 expression in SARS-CoV-2 infection in the *in vitro* model cell line with increased interferon responses, the enhancement was rather limited considering the strong induction levels by enterocytic differentiation. Thus, further studies are needed to dissect the precise effects of IFN-induced ACE2 expression on viral propagation. It would also be interesting to investigate how the ACE2 expression levels are regulated by cytokines or enterocytic differentiation. Upstream regulatory elements and transcription factors for ACE2 have been previously identified [47,48]. However, pro-inflammatory cytokines (TGF-b1 or TNF-a) do not significantly affect the transcriptional activation of ACE2 [47].

Given that the GI symptoms are a major indication of SARS-CoV-2 infection [49], the intestinal brush border cells capable of robust and persistent infection would be a useful tool for understanding the pathogenesis of SARS-CoV-2 in the GI tract.

## Supplementary Material

Supplemental Material
