# Supplementary material for: Robust and persistent SARS-CoV-2 infection in the human intestinal brush border expressing cells
Source: Emerg Microbes Infect. 2020 Oct 3;9(1):2169–79. doi: 10.1080/22221751.2020.1827985 (PMC7580600; doi:10.1080/22221751.2020.1827985)
Supplement: Supplemental Material [file TEMI_A_1827985_SM4707.docx]

**Supplemental materials**

**Figure legends**

Fig. S1. Growth kinetics of SARS-CoV-2 in non-human cells.

The indicated cells were grown on a 48-well plate and infected with 5 MOI of SARS-CoV-2. Viral RNA levels were determined in the media collected at the indicated time points. Data are presented as mean values with error bars showing the standard deviations from three independent experiments. ND, not detected.

Fig. S2. Cytopathic effects of the SARS-CoV-2 infected cells.

The indicated cells were infected with 5 MOI of SARS-CoV-2, and the cell morphologies were observed at 4 dpi.

Fig. S3. The protein expression level of the ACE2 receptor in various cell lines.

ACE2 protein in the indicated cell lysates was detected by Western blotting. The upper panel shows the underexposed blot of ACE2 and lower panel the overexposed blot.

Fig. S4. Infectivity of progeny SARS-CoV-2 from the persistently infected C2BBe1 cells.

1. Schematic diagram of the experimental timeline. Initially, 1 MOI of SARS-CoV-2 was infected into C2BBe1 cells (P0). The cells were passaged 4 times (P1-P4) for a total of 23 days. (B) Vero CCL81 cells grown on a 6-well plate were incubated with or without 100 µl of media collected from the C2BBe1 cells persistently infected with SARS-CoV-2 at 23 dpi. CPEs in the Vero CCL81 cells were observed at 2 dpi.

**Fig. S1**


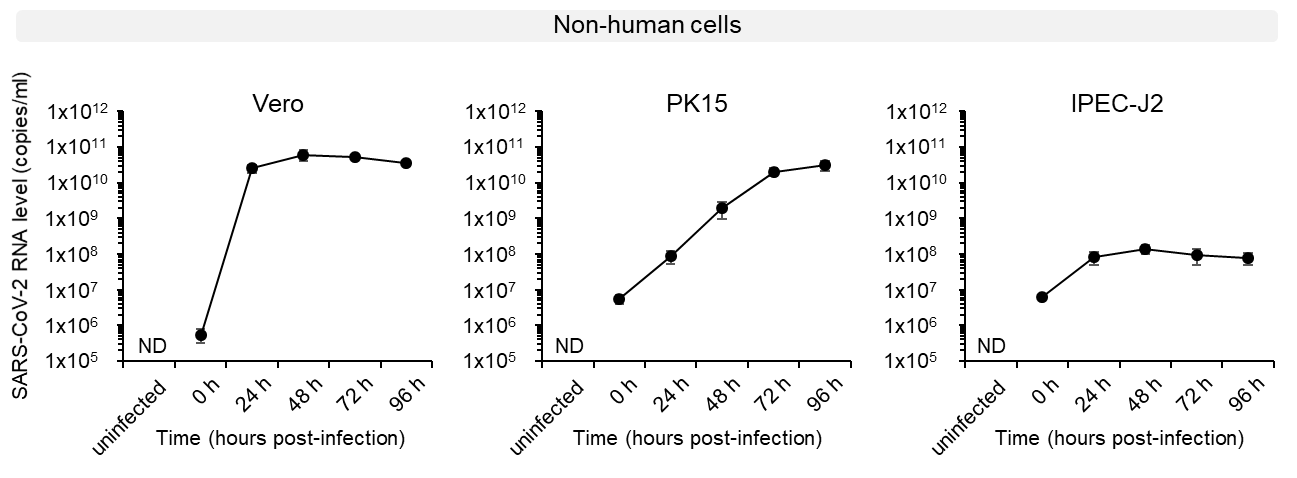


| **Fig. S2** | **Calu-3** |  |
| --- | --- | --- |
|  | **uninfected** | **SARS-CoV-2 (4 dpi)** |


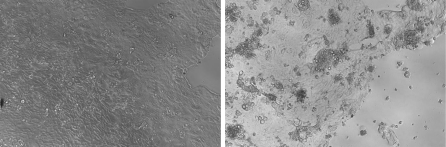


**NCI- H292**


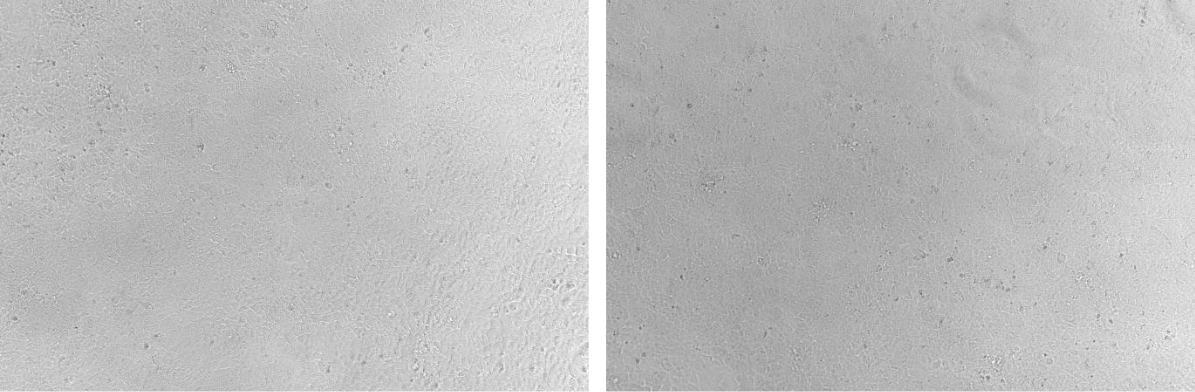


**uninfected** **SARS-CoV-2 (4 dpi)**

**C2BBe1**


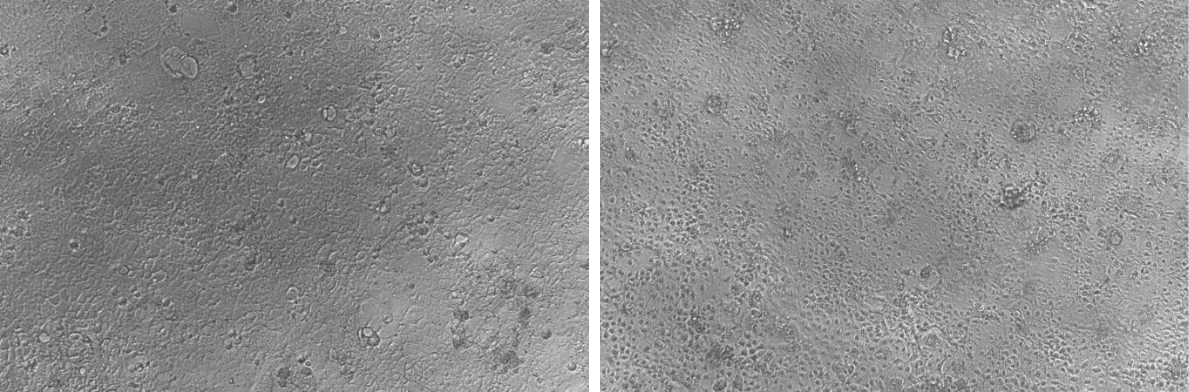


**uninfected** **SARS-CoV-2 (4 dpi)**

**A549**


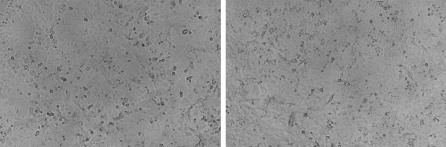


**uninfected** **SARS-CoV-2 (4 dpi)**

**Caco-2**


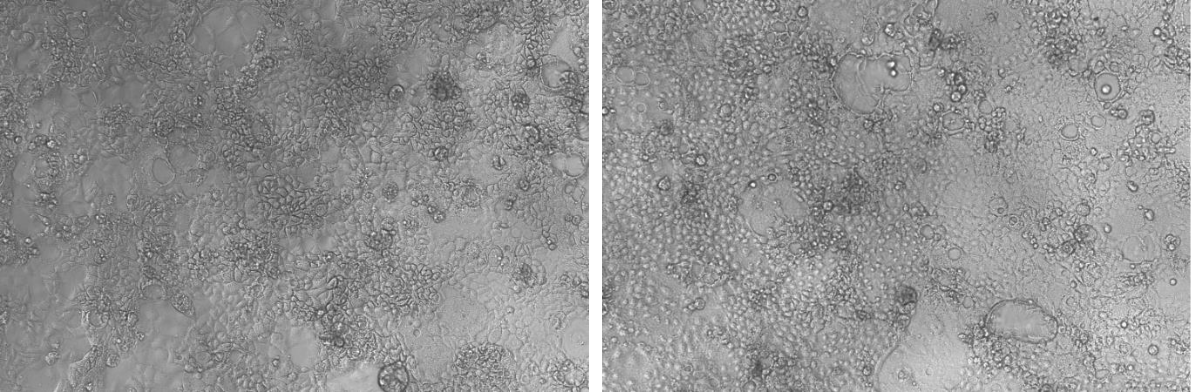


**uninfected** **SARS-CoV-2 (4 dpi)**

**RPMI 2650**


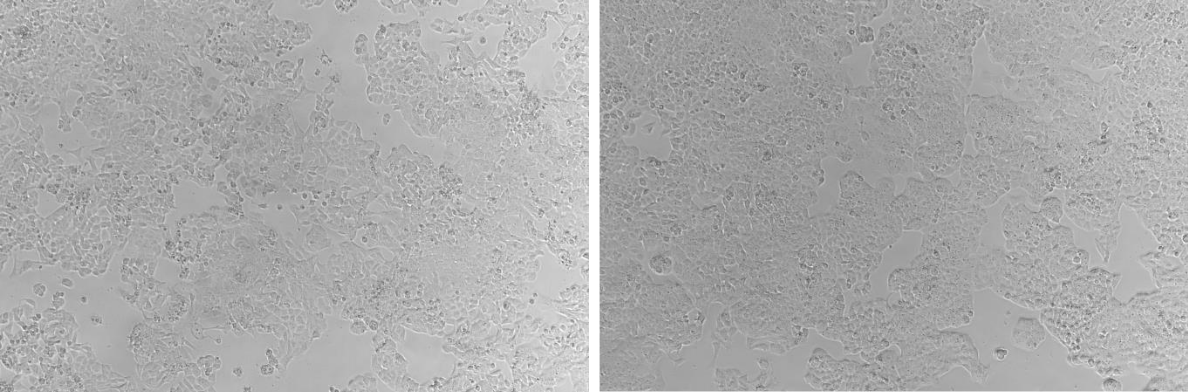


**uninfected** **SARS-CoV-2 (4 dpi)**

**Fig. S2 (continued)**

**Vero**


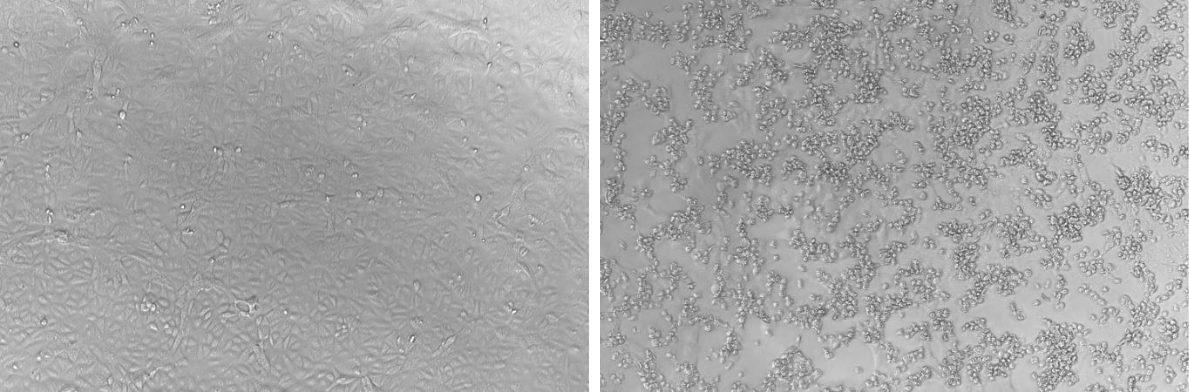


**uninfected** **SARS-CoV-2 (4 dpi)**

**IPEC-J2**


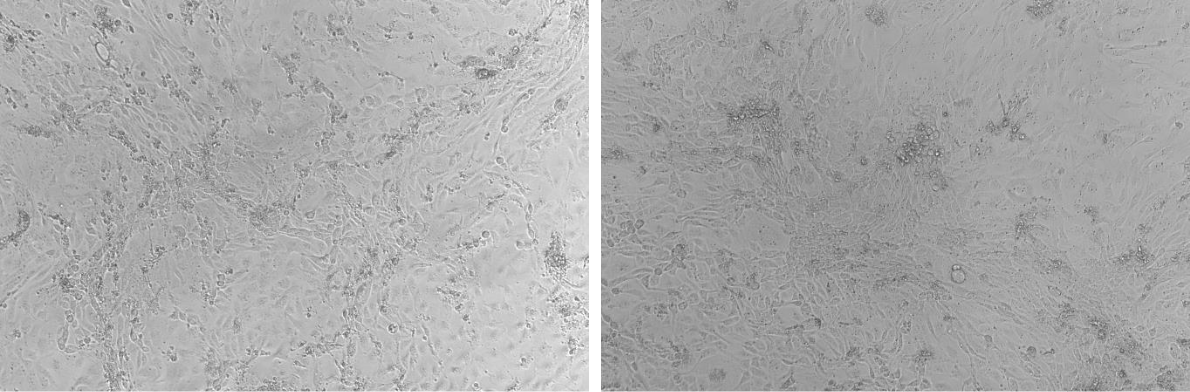


**uninfected** **SARS-CoV-2 (4 dpi)**

**PK-15**


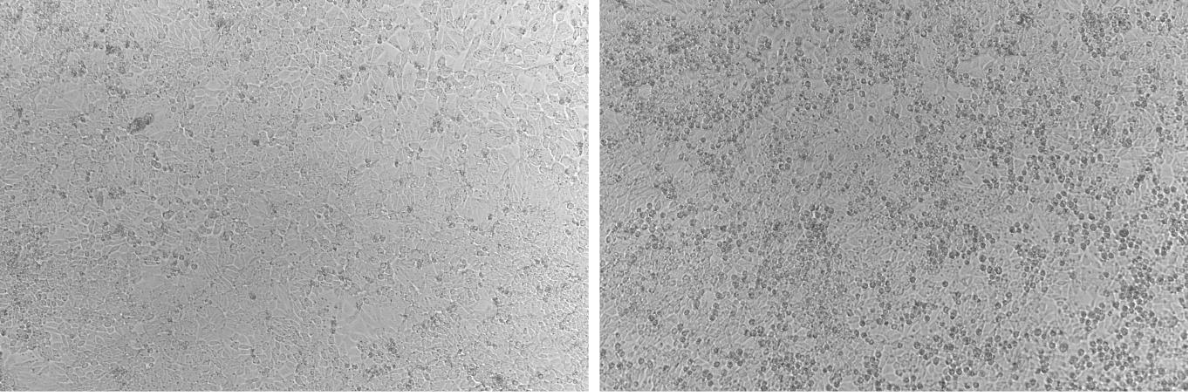


**uninfected** **SARS-CoV-2 (4 dpi)**

**Fig. S3**


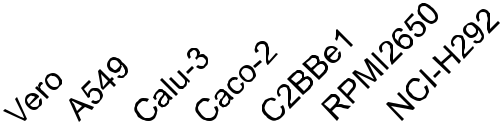


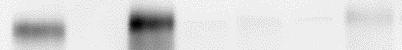
- ACE2 (under)


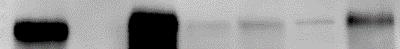
- ACE2 (over)


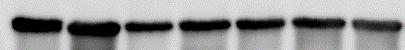
- β-actin

**Fig. S4**


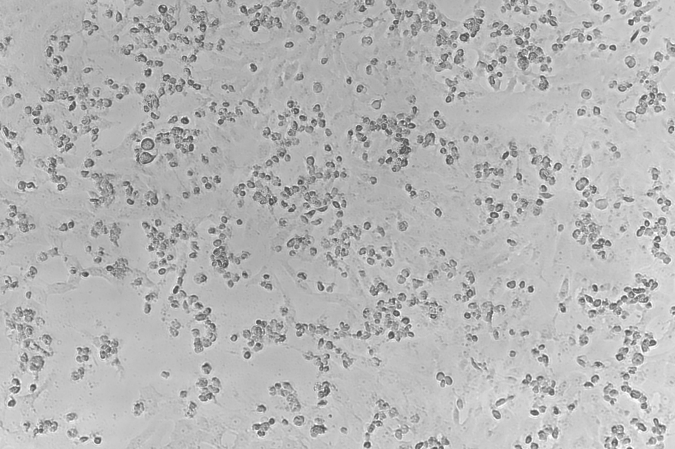

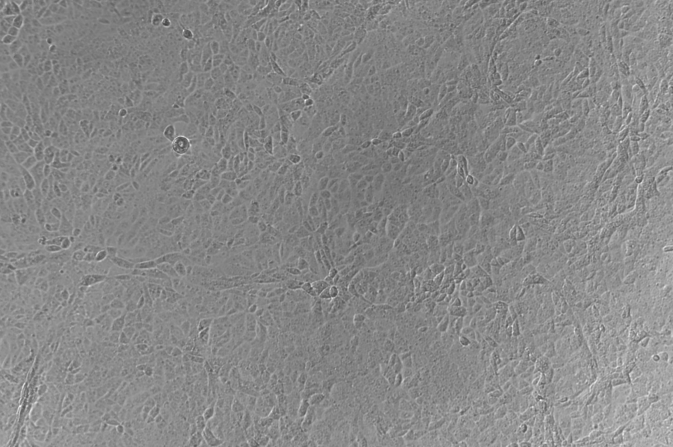


**SARS-CoV-2 from**

**persistently infected**

**C2BBe1 cells (23 dpi)**

**uninfected**

0

3

8

13

18

23

P0

P1

P2

P3

P4

dpi

Passage

Infection

Vero cells

Reinfection

**B**

**A**
